# Evaluation of the Prognostic Value of the Mayo Additive Staging System and Minimal Residual Disease in Newly Diagnosed Multiple Myeloma Patients

**DOI:** 10.1002/cam4.70382

**Published:** 2024-11-04

**Authors:** Yichuan Song, Rui Zhao, Wenxuan Fu, Jing Zhao, Qingtao Wang, Rui Zhang

**Affiliations:** ^1^ Department of Clinical Laboratory, Beijing Chao‐Yang Hospital Capital Medical University Beijing China

**Keywords:** Mayo additive staging system, minimal residual disease, multiple myeloma, prognosis, response to treatment

## Abstract

**Introduction:**

This study aimed to evaluate the prognostic value of the Mayo additive staging system (MASS) and minimal residual disease (MRD) in newly diagnosed multiple myeloma (NDMM) patients.

**Methods:**

A total of 238 NDMM patients were enrolled in Beijing Chaoyang Hospital. Fluorescence in‐situ hybridization and next‐generation flow cytometry were used to examine cytogenetic abnormalities and MRD, respectively. The patients were classified into three groups to compare the effects on progression‐free survival (PFS). Univariate and multivariate analyses were applied to identify the survival‐related factors.

**Results:**

For MASS group, the PFS was significant difference in MASS I, II, and III patients (*p* = 0.0006); the patients with sustained MRD‐negative, non‐sustained MRD‐negative, sustained MRD‐positive, and non‐sustained MRD‐positive were divided into Groups 1, 2, 3, and 4, respectively. The Group 1 patients had superior PFS than Groups 2 and 3 patients (*p* < 0.05), but no difference in PFS was observed for Group 2 versus Group 3, Group 2 versus Group 4, and Group 3 versus Group 4 patients. For the MASS and MRD groups, among Groups 2, 3, and 4, MASS I patients had a superior PFS, while III patients showed the opposite result. Strikingly, no difference in PFS for Group 1 patients regardless of the MASS stage was observed. Despite being in MASS II and III, the PFS of Group 1 patients was longer than those with the other three groups. Response to treatment was an independent prognostic factor for MM patients.

**Conclusion:**

Patients with an accumulation of high‐risk factors and MRD‐positive have poor outcomes. Sustained MRD‐negative may improve high‐risk patients' prognoses.

## 1 Introduction

Multiple myeloma (MM), a bone marrow cancer that stimulates plasma cell growth, causes more than 10% of all hematological malignancies worldwide [1]. Men and the elderly have a high probability of developing MM. The occult development and late diagnosis of MM compromise many patients' life quality [2, 3]. Although new drugs (such as protease inhibitors, immunomodulators, and monoclonal antibodies) have greatly improved longevity, some patients' outcomes remain relatively poor [4].

MM is highly heterogeneous in terms of clinical manifestations, biological characteristics, response to treatment, and clinical outcomes. Accurate risk stratification is very important for guiding treatment decisions. The international staging system (ISS) and revised ISS (R‐ISS) have a wide application in clinical practice. However, both two risk stratification systems have limitations. To address the concerns of these systems, the Mayo additive staging system (MASS) has been proposed based on the five high‐risk factors and provides prognostic value in newly diagnosed multiple myeloma (NDMM) patients [5, 6, 7, 8]. Furthermore, many patients achieving complete remission (CR) eventually relapse due to their minimal residual disease (MRD). The International Myeloma Working Group (IMWG) proposed the response criteria based on MRD assessment [9]. MRD can reflect a patient's remission more deeply than CR and make it easier to determine whether the disease has returned [10]. Simultaneously, MRD has also a substantial impact on the prognosis of MM patients [11, 12, 13].

At present, there is limited research on the prognostic value of the MASS and different MRD statuses among NDMM patients in China. In the study, we collected clinical indicators, exploring the impacts of these indicators on the response to treatment, survival time, and prognosis in 238 NDMM patients.

## 2 Materials and Methods

### 2.1 Patients and Treatment

All patients screened electronic medical records from January 2014 to April 2023 with NDMM and completed Fluorescence in‐situ hybridization (FISH) results in Chao‐yang Hospital. Cases were excluded if they had been diagnosed with plasma cell leukemia or incomplete baselines. Finally, 238 NDMM patients were collected. The 238 NDMM patients included 136 men and 102 women, with a median age of 61 years (range 25–84 years). Patients were treated with various induction, consolidation, and maintenance therapy cycles. The regimens containing VRD (bortezomib, dexamethasone, and cyclophosphamide), BCD (bortezomib, cyclophosphamide, and cyclophosphamide), BDD (bortezomib, lipodoxorubicin, and cyclophosphamide), BD (bortezomib and cyclophosphamide) were used for initial therapy in 35.2%, 19.3%, 13.4%, and 5.0% of the patients, respectively. Of all NDMM patients, 88 patients (37.0%) have received ASCT after induction therapy. About half of the patients received Lenalidomide‐based regimens, and others received no or proteasome inhibitor‐based maintenance therapy. The degree of remission was evaluated at the last follow‐up. The study was performed in line with the principles of the Declaration of Helsinki. A waiver was granted by the Beijing Chaoyang Hospital's ethical committee (2022‐ke‐48).

### 2.2 Fluorescence In‐Situ Hybridization

Because FISH is the gold standard method for detecting MM cytogenetic abnormalities (CAs) [14], we employed it to identify interphase cells after purifying plasma cells expressing CD138 as previously described [4]. Technical thresholds were determined for each probe using isolated CD138 expression from patients without MM, based on the same method as used with MM patients. Thresholds were assessed after counting 200 cells for each negative sample and established by a mean “mean + 3SD” calculation. The threshold for positive results was used: 1.11% for t(4;14), 0.77% for t(14;16), 6.87% for gain/amp 1q21 and 6.09% for del(17p).

The MASS was established on the five factors: high‐risk IgH translocations [t(4;14) and t(14;16)], gain/amp 1q21, del(17p), ISS III, and/or elevated lactate dehydrogenase (LDH) [5, 6, 7, 8]. Each factor was scored as 1 point, and patients with 0, 1, or ≥ 2 points were divided into MASS I, II, or III, respectively.

### 2.3 Next‐Generation Flow Cytometry

This method can be used to evaluate MRD by identifying any leftover tumor cells on bone marrow aspirates. As previously stated [15], antibodies for CD38, CD138, CD19, CD56, CD27, CD81, CD117, CD45, cκappa, and cλambda were chosen. A total of 10 million nucleated cells were collected, and the number of abnormal plasma cells was determined through the antigen expression of normal and abnormal plasma cells.

In total, 166 patients was performed for MRD assessment, and the median follow‐up duration was 25 months. The patients was evaluated for MRD assessment after intensification/consolidation until the last follow‐up. The NDMM patients were divided into four groups based on MRD results with 10^−5^ as the standard: Group 1 (sustained MRD‐negative, MRD < 10^−5^ continuously for more than 12 months), Group 2 (non‐sustained MRD‐negative, MRD < 10^−5^ not continuously for 12 months or transitioned from positive to negative after treatment), Group 3 (sustained MRD‐positive, MRD > 10^−5^ continuously for more than 12 months), and Group 4 (non‐sustained MRD‐positive, MRD > 10^−5^ not continuously for 12 months or transitioned from negative to positive after treatment).

### 2.4 Statistical Analysis

The progression‐free survival (PFS) of patients was followed through April 30, 2023, in this study. PFS was defined as the period from diagnosis to progression, relapse, or last follow‐up.

The data was analyzed using GraphPad Prism 8 and IBM SPSS Statistics 26.0. The categorical variables were analyzed by Chi‐squared tests and Fisher's exact tests, while continuous variables were analyzed by Kruskal–Wallis *H* tests, Mann–Whitney *U* tests, and *t*‐tests. PFS was assessed by the Kaplan–Meier method and compared the differences between groups using the log‐rank test. In multivariate analysis, the Cox proportional hazards model comprised PFS prognostic factors with a significant *p*‐value (*p* < 0.1). *p* < 0.05 was considered statistically significant.

## 3 Results

### 3.1 Clinical Characteristics of NDMM Patients

**FIGURE 1:**
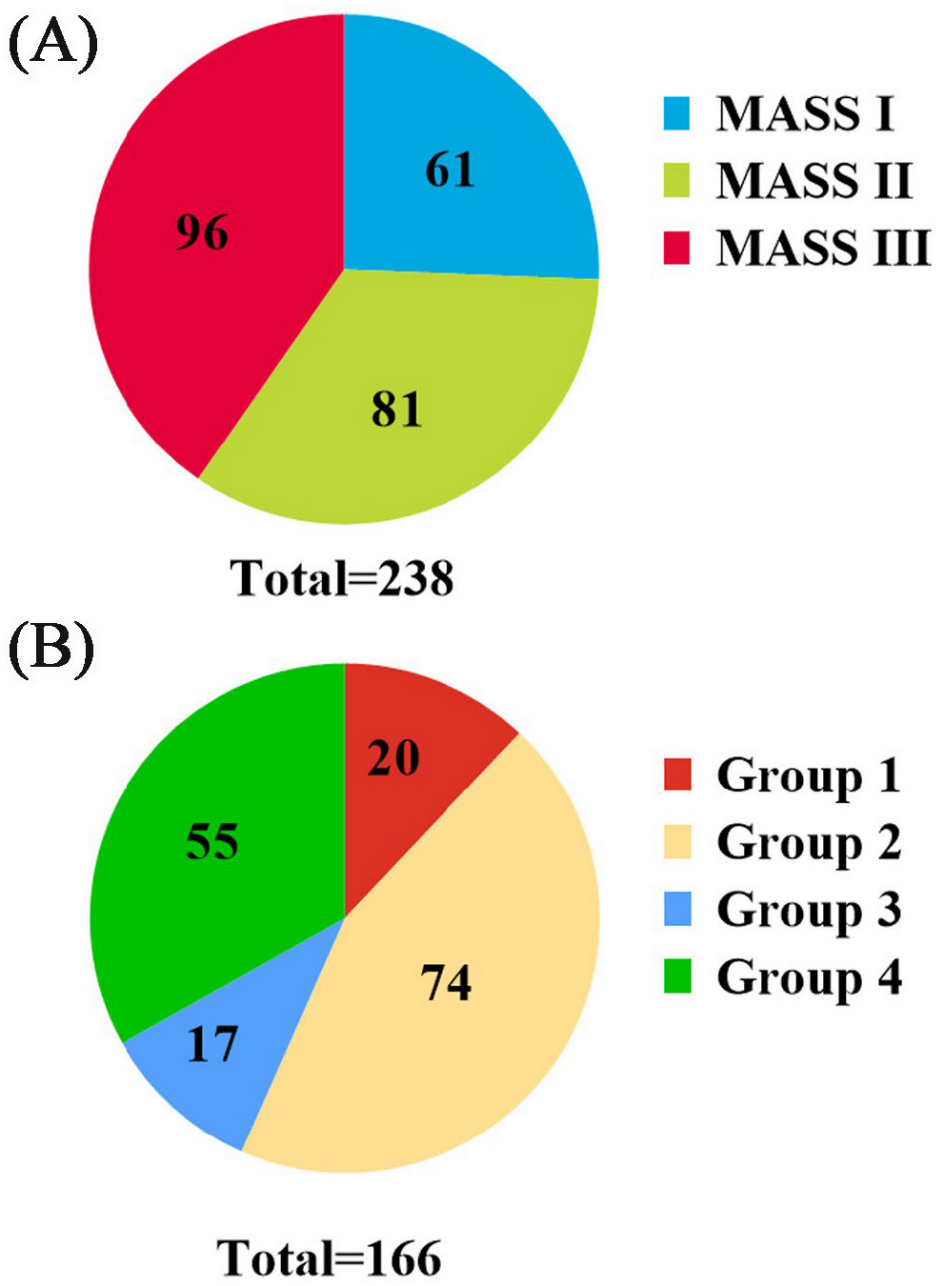
The distribution of the MASS (A) and MRD (B) among 238 NDMM patients. Group 1: Sustained MRD‐negative; Group 2: Non‐sustained MRD‐negative; Group 3: Sustained MRD‐positive; Group 4: Non‐sustained MRD‐positive.

According to the MASS, 61 (25.6%), 81 (34.0%), and 96 (40.4%) patients were classified into MASS I, II, and III, respectively (Figure 1A). Owing to the higher proportion of patients with gain/amp 1q21 (45.2%), there were more MASS II and III patients compared to I patients. MASS II and III patients had a higher proportion of ISS III (*p* < 0.0001), elevated lactate dehydrogenase (LDH) (*p* < 0.0001), elevated creatinine (*p* = 0.0005), and elevated beta‐2‐microglobulin (β2‐MG) (*p* < 0.0001) than I patients. However, lower albumin (ALB) (*p* = 0.0080), albumin to globulin ratio (A/G) (*p* = 0.0144), platelet (PLT) (*p* = 0.0007), and hemoglobin (Hb) (*p* < 0.0001) levels were observed in MASS II and III patients compared with I patients. Age, sex, Durie‐Salmon stage, immunoglobulin isotypes, extramedullary disease, overall response rate (ORR), plasma cells on bone marrow, globulin (GLB), and calcium (Ca) were similar among the three groups.

Among the 238 NDMM patients, 166 patients were evaluated for MRD results, with 20 (12.0%) in Group 1, 74 (44.6%) in Group 2, 17 (10.2%) in Group 3, and 55 (33.2%) in Group 4 (Figure 1B). There were no significant differences in most characteristics among four groups, except for sex (*p* = 0.0268) and ORR (*p* = 0.0007). Table 1 summarizes the detailed clinical characteristics.

**TABLE 1:** Baseline characteristics of MM patients based on the MASS and MRD statuses.

Characteristics	Groups based on the MASS				Groups based on MRD statuses				
	MASS I (*N* = 61)	MASS II (*N* = 81)	MASS III (*N* = 96)	*p*	Group 1^c^ (*N* = 20)	Group 2^c^ (*N* = 74)	Group 3^c^ (*N* = 17)	Group 4^c^ (*N* = 55)	*p*
Age				0.7523					0.8544
> 65	19 (31.1%)	27 (33.3%)	27 (28.1%)		6 (30.0%)	18 (24.3%)	3 (17.6%)	14 (25.5%)	
≤ 65	42 (68.9%)	54 (66.7%)	69 (71.9%)		14 (70.0%)	56 (75.7%)	14 (82.4%)	41 (74.5%)	
Sex (male/female)	37/24 (1.54)	42/39 (1.08)	57/39 (1.46)	0.4895	11/9 (1.22)	32/42 (0.76)	11/6 (1.83)	38/17 (2.24)	0.0268*
ISS stage				< 0.0001***					0.4416
I + II	61 (100.0%)	46 (56.8%)	17 (17.7%)		9 (45.0%)	44 (59.5%)	8 (47.1%)	26 (47.3%)	
III	0	35 (43.2%)	79 (82.3%)		11 (55.0%)	30 (40.5%)	9 (52.9%)	29 (52.7%)	
Durie‐Salmon stage				0.1972					0.9138
I + II	15 (24.6%)	11 (13.6%)	15 (15.6%)		4 (20.0%)	14 (18.9%)	3 (17.6%)	8 (14.5%)	
III	46 (75.4%)	70 (86.4%)	81 (84.4%)		16 (80.0%)	60 (81.1%)	14 (82.4%)	47 (85.5%)	
Immunoglobulin isotype				0.4281					0.6345
Immunoglobulin G (IgG)	28 (45.9%)	39 (48.1%)	40 (41.7%)		8 (40.0%)	33 (44.6%)	5 (29.4%)	22 (40.0%)	
Light chain type	21 (34.4%)	25 (30.9%)	26 (27.1%)		6 (30.0%)	27 (36.5%)	5 (29.4%)	18 (32.7%)	
Others^a^	12 (19.7%)	17 (21.0%)	30 (31.2%)		6 (30.0%)	14 (18.9%)	7 (41.2%)	15 (27.3%)	
Extramedullary disease				0.8747					0.6508
Yes	6 (9.8%)	10 (12.3%)	10 (10.4%)		1 (5.0%)	9 (12.2%)	3 (17.6%)	8 (14.5%)	
No	55 (90.2%)	71 (87.7%)	86 (89.6%)		19 (95.0%)	65 (87.8%)	14 (82.4%)	47 (85.5%)	
Response to treatment				0.1620					0.0007***
Overall response rate (ORR)^b^	48 (78.7%)	67 (82.7%)	68 (70.8%)		20 (100.0%)	71 (95.9%)	12 (70.6%)	43 (78.2%)	
Plasma cells on bone marrow (%)				0.0977					0.8320
≥ 60%	9 (14.8%)	12 (14.8%)	25 (26.0%)		6 (30.0%)	18 (24.3%)	4 (23.5%)	11 (20.0%)	
< 60%	52 (85.2%)	69 (85.2%)	71 (74.0%)		14 (70.0%)	56 (75.7%)	13 (76.5%)	44 (80.0%)	
Albumin (ALB) (g/L)				0.0080**					0.7131
> 35 g/L	42 (68.9%)	49 (60.5%)	43 (44.8%)		13 (65.0%)	43 (58.1%)	8 (47.1%)	30 (54.5%)	
≤ 35 g/L	19 (31.1%)	32 (39.5%)	53 (55.2%)		7 (35.0%)	31 (41.9%)	9 (52.9%)	25 (45.5%)	
Globulin (GLB) (g/L)				0.0785					0.9958
> 40 g/L	28 (45.9%)	40 (49.4%)	60 (62.5%)		11 (55.0%)	41 (55.4%)	9 (52.9%)	31 (56.4%)	
≤ 40 g/L	33 (54.1%)	41 (50.6%)	36 (37.5%)		9 (45.0%)	33 (44.6%)	8 (47.1%)	24 (43.6%)	
Albumin to Globulin ratio (A/G)	1.2	0.9	0.7	0.0144*	0.85	0.90	0.80	0.70	0.9201
Lactate dehydrogenase (LDH) (U/L)				< 0.0001***					0.4611
> 222 U/L	0	76 (93.8%)	66 (68.8%)		1 (5.0%)	15 (20.3%)	3 (17.6%)	10 (18.2%)	
≤ 222 U/L	61 (100.0%)	5 (6.2%)	30 (31.2%)		19 (95.0%)	59 (79.7%)	14 (82.4%)	45 (81.8%)	
Creatinine (Cre) (umol/L)				0.0005***					0.5824
> 177 umol/L	2 (3.3%)	16 (19.8%)	27 (28.1%)		4 (20.0%)	10 (13.5%)	4 (23.5%)	12 (21.8%)	
≤ 177 umol/L	59 (96.7%)	65 (80.2%)	69 (71.9%)		16 (80.0%)	64 (86.5%)	13 (76.5%)	43 (78.2%)	
Calcium (Ca) (mmol/L)	2.30	2.27	2.22	0.2390	2.38	2.24	2.29	2.24	0.5546
Platelet (PLT) (×10^9^/L)				0.0007***					0.9982
> 150 × 10^9^/L	54 (88.5%)	59 (72.8%)	58 (60.4%)		15 (75.0%)	55 (74.3%)	13 (76.5%)	41 (74.5%)	
≤ 150 × 10^9^/L	7 (11.5%)	22 (27.2%)	38 (39.6%)		5 (25.0%)	19 (25.7%)	4 (23.5%)	14 (25.5%)	
Hemoglobin (Hb) (g/L)				< 0.0001***					0.3713
> 100 g/L	42 (68.9%)	29 (35.8%)	21 (21.9%)		7 (35.0%)	29 (39.2%)	3 (17.6%)	22 (40.0%)	
≤ 100 g/L	19 (31.1%)	52 (64.2%)	75 (78.1%)		13 (65.0%)	45 (60.8%)	14 (82.4%)	33 (60.0%)	
Beta‐2‐microglobulin (β2‐MG)				< 0.0001***					0.1738
> 5.5 mg/L	4 (6.6%)	30 (37.0%)	69 (71.9%)		11 (55.0%)	27 (36.5%)	8 (47.1%)	30 (54.5%)	
≤ 5.5 mg/L	57 (93.4%)	51 (63.0%)	27 (28.1%)		9 (45.0%)	47 (63.5%)	9 (52.9%)	25 (45.5%)	

^a^ Others: The number of patients with Immunoglobulin A (IgA), Immunoglobulin D (IgD), and non‐secretory subtypes.

^b^ Overall response rate (ORR) was defined as partial response (PR), very good partial response (VGPR), complete response (CR), and stringent complete response (sCR).

^c^ Group 1: Sustained MRD‐negative; Group 2: Non‐sustained MRD‐negative; Group 3: Sustained MRD‐positive; and Group 4: Non‐sustained MRD‐positive.

* *p* < 0.05, indicating significant differences among groups.

** *p* < 0.01, indicating significant differences among groups.

*** *p* < 0.001, indicating significant differences among groups.

### 3.2 Survival Analysis

**FIGURE 2:**
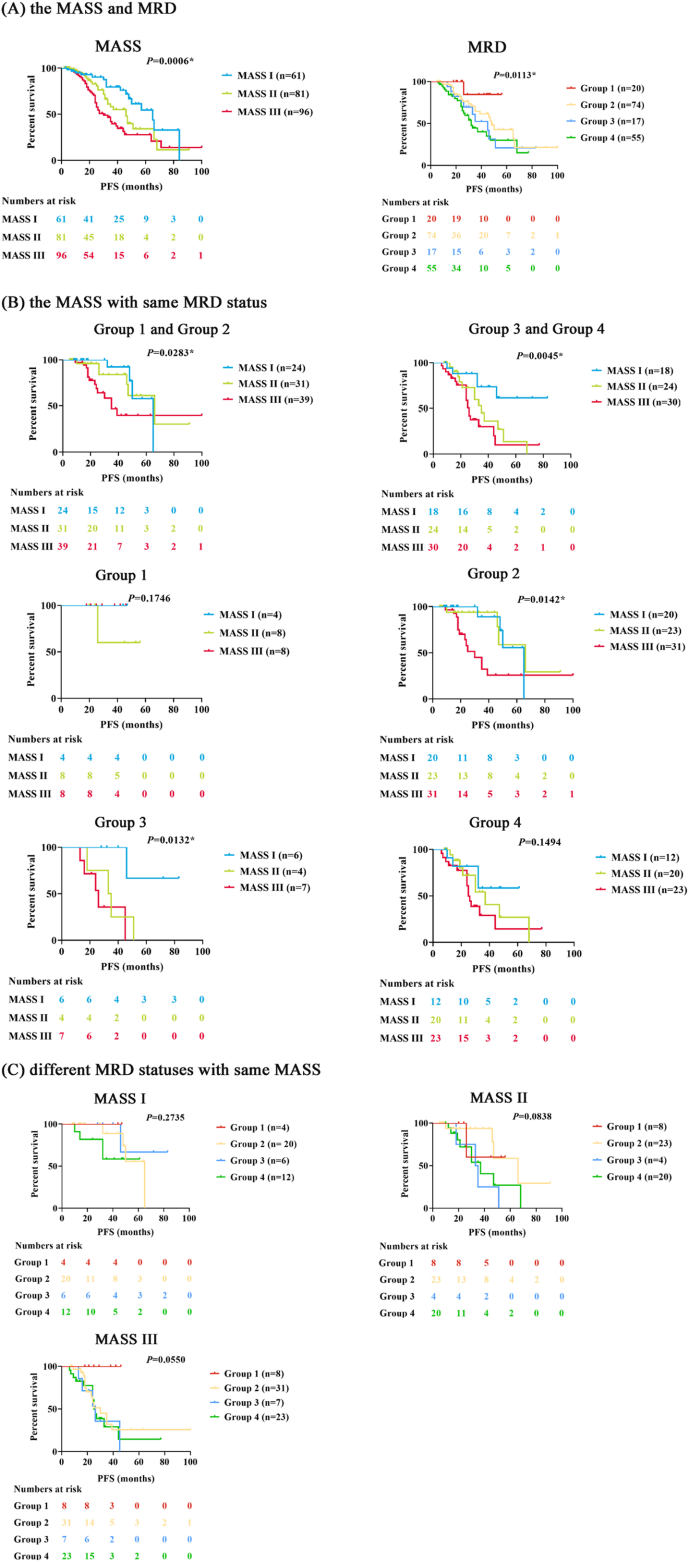
The PFS for NDMM patients based on the MASS and MRD statuses. (A) PFS in the patients with the MASS and different MRD statuses; (B) PFS in the MASS I, II, and III patients with different MRD statuses; (C) PFS in the different MRD statuses patients with the same MASS stage. Group 1: Sustained MRD‐negative; Group 2: Non‐sustained MRD‐negative; Group 3: Sustained MRD‐positive; Group 4: Non‐sustained MRD‐positive; Group 1 and Group 2: MRD‐negative (sustained MRD‐negative and non‐sustained MRD‐negative); Group 3 and Group 4: MRD‐positive (sustained MRD‐positive and non‐sustained MRD‐positive).

The median follow‐up duration of our cohort was 29 months. For MASS groups, there were significant differences in PFS for MASS I versus II (*p* = 0.0476), I versus III (*p* = 0.0003), and II versus III (*p* = 0.0468) patients. PFS was considerably prolonged for Group 1 patients compared to Group 2 (*p* = 0.0455), Group 3 (*p* = 0.0112), and Group 4 (*p* = 0.0022) patients. However, the Group 3 and 4 patients had a similar PFS, and the same as Group 2 versus Group 3 and Group 2 versus Group 4 patients (as shown in Figure 2A).

To further understand the combined impact of the MASS stages and different MRD statuses, we first implemented a separate analysis with three MASS stage patients who had the same MRD status. Among the patients with MRD‐negative (Group 1 and 2), the PFS showed significant differences between MASS I and III patients (*p* = 0.0301). There seemed to be a trend toward similar PFS for I versus II and II versus III patients. For MRD‐positive (Group 3 and 4) patients, there were significant differences in PFS for MASS I versus II (*p* = 0.0279) and I versus III patients (*p* = 0.0018), but the PFS was similar for II versus III patients. Furthermore, for Group 1 and 4 patients, the PFS of the pairwise comparisons between the two of the three stages indicated no significant differences, respectively. For Group 2, MASS II patients showed superior PFS than III patients (*p* = 0.0199), but not significantly different from I patients. The significant difference in PFS was shown between MASS I and III patients (*p* = 0.0270). Conversely, no difference in PFS for II versus III patients in Group 3 was found, but I patients were associated with better PFS compared to II (*p* = 0.0329) and III patients (*p* = 0.0054, as shown in Figure 2B).

Then, we also evaluated the different MRD statuses among patients with the same MASS stage. In the MASS I patients, there were no differences in PFS between any of the pairwise comparisons. In the MASS II patients, the Group 2 patients showed better PFS compared to those in Group 3 (*p* = 0.0255). Among the other pairwise comparison groups, there were a trend toward similar PFS. In the MASS III patients, the differences in PFS were significant for Group 1 versus Group 2 (*p* = 0.0127) and Group 1 versus Group 3 (*p* = 0.0123) patients, but not for patients among the other pairwise comparison groups (as shown in Figure 2C).

### 3.3 Univariate Analysis and Multivariate Analysis

In univariate analysis (as shown in Table 2), MASS stage, ISS stage, response to treatment, PLT, and β2‐MG for the MASS group, MRD statuses, ISS stage, response to treatment, PLT, and β2‐MG for the MRD group, MASS and MRD statuses, ISS stage, response to treatment, PLT, and β2‐MG for MASS and MRD group were significant impact on PFS. The factors related to PFS in univariate analysis were then implemented in multivariate analysis (as shown in Table 3). MASS stage, response to treatment (< VGPR), and PLT ≤ 150 × 10^9^/L were significantly associated with poor PFS for the MASS group. Significant prognostic factors in PFS for the MRD group were ISS III and response to treatment (< VGPR). For the MASS and MRD group, MASS and MRD statuses and response to treatment (< VGPR) were independent factors for inferior outcomes.

**TABLE 2:** Impact of baseline characteristics on PFS in univariate analysis.

Factors	Groups based on the MASS stage		Groups based on MRD statuses		Groups based on the MASS stage and MRD statuses	
	HR (95% CI)	*p*	HR (95% CI)	*p*	HR (95% CI)	*p*
MASS stage	1.665 (1.272–2.180)	< 0.001***	/	/	/	/
MRD statuses	/	/	1.478 (1.158–1.886)	0.002**	/	/
MASS and MRD statuses	/	/	/	/	1.216 (1.118–1.322)	< 0.001***
Age (> 65 vs. ≤ 65)	1.210 (0.770–1.902)	0.408	0.974 (0.535–1.774)	0.932	0.974 (0.535–1.774)	0.932
Sex (male vs. female)	1.482 (0.967–2.270)	0.071	1.475 (0.879–2.474)	0.141	1.475 (0.879–2.474)	0.141
Durie‐Salmon stage (DS III vs. DS I and II)	1.146 (0.657–1.997)	0.631	1.151 (0.583–2.269)	0.686	1.151 (0.583–2.269)	0.686
ISS stage (ISS III vs. ISS I and II)	1.936 (1.273–2.943)	0.002**	2.110 (1.261–3.533)	0.005**	2.110 (1.261–3.533)	0.005**
Immunoglobulin isotype						
Others^a^ versus IgG	0.908 (0.524–1.574)	0.731	0.987 (0.504–1.932)	0.970	0.987 (0.504–1.932)	0.970
Light chain type versus IgG	1.368 (0.859–2.179)	0.187	1.511 (0.849–2.690)	0.160	1.511 (0.849–2.690)	0.160
Extramedullary disease (yes vs. no)	1.374 (0.779–2.423)	0.272	1.600 (0.848–3.019)	0.147	1.600 (0.848–3.019)	0.147
Response to treatment (< VGPR vs. ≥ VGPR^b^)	4.497 (2.878–7.027)	< 0.001***	2.993 (1.790–5.004)	< 0.001***	2.993 (1.790–5.004)	< 0.001***
Plasma cells on bone marrow (> 60% vs. ≤ 60%)	0.695 (0.348–1.388)	0.302	0.741 (0.352–1.562)	0.431	0.741 (0.352–1.562)	0.431
Albumin (ALB) (> 35 vs. ≤ 35 g/L)	1.080 (0.711–1.639)	0.719	1.001 (0.601–1.669)	0.996	1.001 (0.601–1.669)	0.996
Globulin (GLB) (> 40 vs. ≤ 40 g/L)	0.940 (0.623–1.420)	0.769	0.922 (0.557–1.527)	0.753	0.922 (0.557–1.527)	0.753
A/G	1.082 (0.812–1.443)	0.591	1.072 (0.759–1.513)	0.693	1.072 (0.759–1.513)	0.692
Lactate dehydrogenase (LDH) (> 222 vs. ≤ 222 U/L)	1.464 (0.823–2.604)	0.195	1.715 (0.923–3.188)	0.088	1.017 (0.406–2.549)	0.971
Creatinine (Cre) (> 177 vs. ≤ 177 μmol/L)	1.217 (0.725–2.043)	0.457	0.938 (0.488–1.806)	0.849	0.938 (0.488–1.806)	0.849
Calcium (Ca)	1.203 (0.601–2.406)	0.602	0.882 (0.317–2.453)	0.810	0.880 (0.316–2.448)	0.807
Platelet (PLT) (≤ 150 vs. > 150 × 10^9^/L)	1.923 (1.214–3.045)	0.005**	1.988 (1.141–3.463)	0.015*	1.988 (1.141–3.463)	0.015*
Hemoglobin (Hb) (> 100 vs. ≤ 100 g/L)	0.832 (0.544–1.273)	0.396	0.635 (0.365–1.104)	0.107	0.635 (0.365–1.104)	0.107
Beta‐2‐microglobulin (β_2_‐MG) (> 5.5 vs. ≤ 5.5 mg/L)	1.698 (1.123–2.568)	0.012*	1.741 (1.047–2.896)	0.033*	1.781 (1.072–2.959)	0.026*

*Note:* / indicating the factors were not included in univariate analysis.

Abbreviation: HR, hazard ratio.

^a^ Others: The number of patients with IgA, IgD, and non‐secretory subtypes.

^b^ ≥ VGPR was defined as very good partial response (VGPR), complete response (CR), and stringent complete response (sCR).

* *p* < 0.05, indicating significant differences among groups.

** *p* < 0.01, indicating significant differences among groups.

*** *p* < 0.001, indicating significant differences among groups.

**TABLE 3:** Impact of baseline characteristics on PFS in multivariate analysis.

Factors	Groups based on the MASS stage		Groups based on MRD statuses		Groups based on the MASS stage and MRD statuses	
	HR (95%CI)	*p*	HR (95% CI)	*p*	HR (95% CI)	*p*
MASS stage	1.620 (1.236–2.122)	< 0.001***	/	/	/	/
MASS and MRD statuses	/	/	/	/	1.210 (1.117–1.312)	< 0.001***
ISS stage (ISS III vs. ISS I and II)	/	/	2.528 (1.493–4.282)	0.001**		
Response to treatment (< VGPR vs. ≥ VGPR^a^)	5.094 (3.228–8.039)	< 0.001***	3.437 (2.035–5.804)	< 0.001***	3.000 (1.775–5.072)	< 0.001***
PLT (≤ 150 vs. > 150 × 10^9^/L)	2.047 (1.275–3.286)	0.003**	/	/	/	/

*Note:* / Indicating the factors were not included in multivariate analysis or no significant difference.

Abbreviation: HR: hazard ratio.

^a^ ≥ VGPR was defined as very good partial response (VGPR), complete response (CR), and stringent complete response (sCR).

** *p* < 0.01, indicating significant differences among groups.

*** *p* < 0.001, indicating significant differences among groups.

## 4 Discussion

MM remains heterogeneous so that the survival outcomes of patients vary greatly, which suggests the importance of risk stratification to discriminate between different risk groups of patients and choose optimal treatment strategies. The MASS considers the compounding effects of multiple adverse factors to overcome the drawbacks of ISS and R‐ISS [5, 6, 7, 8]. In addition, MRD has been accepted as the most important prognostic factor and is associated with the survival of patients. In this study, we analyzed the prognostic value of the MASS and different MRD statuses in NDMM patients. Our findings demonstrate that the MASS can serve as an important tool to distinguish the different risk levels of NDMM patients effectively. Furthermore, attaining sustained MRD‐negative status may lead to a superior outcome in patients with the coexistence of high‐risk factors.

We chose some MM‐related indicators based on the IMWG diagnostic criteria and references [3, 16] to compare the differences between the different groups. Limited evidence suggests that certain primary cytogenetic anomalies and secondary cytogenetic anomalies are linked with clinical features. For example, t(4;14) is linked to higher β2‐MG levels and advanced disease stage [17], and t(14;16) has been linked to renal dysfunction [18]. Gain 1q21 is relevant to advanced disease stage, higher β2‐MG levels and lower Hb levels [19], as well as the patients with del(17p) were presented with higher β2‐MG levels and lower Hb levels [20]. The high‐risk cytogenetic abnormalities (IgH translocations, gain/amp 1q, and chromosome 17 abnormalities) may result in more severe clinical characteristics for MASS II and III patients.

Previous studies have revealed [5, 6, 7, 8] that MASS I, II, and III patients experienced significantly different PFS. In our study, the median PFS of MASS II patients was considerably shorter than that of I, but more prolonged than that of III, which was consistent with the previous studies. Response to treatment, MRD statuses, CAs, R‐ISS stage, ISS stage, and LDH were independently associated with PFS [21, 22, 23, 24, 25, 26, 27, 28]. We found that the MASS stage, response to treatment < VGPR, and PLT ≤ 150 × 10^9^/L were independent risk factors for the MASS group. Tumor burden has become an important factor for outcomes in MM patients. PLT in MM patients was considerably decreased with the ISS stage [29], which may be linked to disease burden/bone marrow (BM) reserve and changes in the BM microenvironment, leading to worse PFS [29, 30]. For MRD groups, ISS stage and response to treatment (< VGPR) were independent risk factors. For the MASS and MRD group, MASS and MRD statuses and response to treatment (< VGPR) were independent factors for inferior outcomes. Only response to treatment < VGPR was an independent risk factor for the three groups. Depth of response is associated with the outcomes of MM patients. The clonal plasma cells of patients achieving ≥ VGPR were dramatically decreased, thereby reducing the tumor burden. Thus, the higher CR or CR plus VGPR rate translated to a longer PFS [31].

MRD detection has grown increasingly important for management in MM patients. Numerous studies [21, 22, 23, 24, 32, 33] have shown that MRD‐negative patients tend to live longer than MRD‐positive patients. In the study of longitudinally evaluated MRD, the sustained MRD‐negative status confers a superior prognosis than three patterns (MRD positive to negative, MRD negative to positive, and persistent MRD positive) [33]. Similarly, another study [34] reported the best PFS was in patients with persistent MRD negative, followed by MRD positive to MRD negative and persistent MRD positive patients. According to a Chinese study [35], patients with sustained MRD negative had a longer PFS than those with loss of MRD negativity and persistent MRD positive. Consistent with previous studies, we showed sustained MRD‐negative had a significantly longer PFS, and sustained and non‐sustained MRD‐positive patients had similarly poor outcomes. However, the median PFS of sustained MRD‐positive patients (45 months) was longer than non‐sustained MRD‐positive patients (32 months), indicating sustained MRD‐positive was not absolutely the adverse outcome and might be correlated with the long‐term stability of disease. Thus, monitoring MRD regularly is important, which contributes to early intervention for non‐sustained MRD statuses patients.

The prognostic utility of the MASS was proved in age groups, renal function groups, and therapeutic regimens groups, but not validated in different MRD statuses groups. Through the retrospective analysis of 166 NDMM patients, we assessed the prognostic value of the MASS among subgroups with different MRD statuses. Our findings found there was no difference in PFS among the MASS I, II, and III patients with sustained MRD‐negative. However, among patients with non‐sustained MRD‐negative, sustained and non‐sustained MRD‐positive, MASS I patients presented a better PFS, yet III patients experienced poor PFS. Besides, sustained MRD‐negative patients had also prolonged PFS compared to patients with the other three MRD statuses even in the MASS II and III patients. These results indicated that attaining sustained MRD‐negative may improve the outcomes and become the potential treatment endpoint for MASS II and III patients.

This study had some limitations. Firstly, this was a single‐center retrospective study. Secondly, the induction, consolidation, and maintenance therapy were varied, which may lead to potential bias on response to treatment and survival. Thirdly, limited patients were available in each subgroup of the MASS and MRD group in the analyses comparing PFS. Thus, the cohort needs to be expanded to confirm these findings. Despite these limitations, our results proved the prognostic utility of the MASS, and showed MASS II and III patients may benefit from sustained MRD‐negative. Moreover, we revealed the independent high prognostic impact of response to treatment < VGPR.

## Author Contributions

**Yichuan Song:** conceptualization (lead), data curation (lead), formal analysis (lead), methodology (lead), writing – original draft (lead). **Rui Zhao:** data curation (supporting). **Wenxuan Fu:** data curation (supporting). **Jing Zhao:** data curation (supporting). **Qingtao Wang:** supervision (equal), writing – review and editing (equal). **Rui Zhang:** funding acquisition (lead), supervision (equal), writing – review and editing (equal).

## Ethics Statement

The study was performed in line with the principles of the Declaration of Helsinki. A waiver was granted by the Beijing Chaoyang Hospital's ethical committee (2022‐ke‐48).

## Conflicts of Interest

The authors declare no conflicts of interest.

## Data Availability

The data that support the findings of this study are available from the corresponding author, upon reasonable request.
